# Sensitive Detection and Simultaneous Discrimination of Influenza A and B Viruses in Nasopharyngeal Swabs in a Single Assay Using Next-Generation Sequencing-Based Diagnostics

**DOI:** 10.1371/journal.pone.0163175

**Published:** 2016-09-22

**Authors:** Jiangqin Zhao, Jikun Liu, Sai Vikram Vemula, Corinna Lin, Jiying Tan, Viswanath Ragupathy, Xue Wang, Christelle Mbondji-wonje, Zhiping Ye, Marie L. Landry, Indira Hewlett

**Affiliations:** 1 DETTD/OBRR/CBER, Food and Drug Administration, Silver Spring, MD, 20993, United States of America; 2 DVP/OVRR/CBER, Food and Drug Administration, Silver Spring, MD, 20993, United States of America; 3 Department of Laboratory Medicine, Yale University School of Medicine, New Haven, CT, 06520, United States of America; Oklahoma State University, UNITED STATES

## Abstract

Reassortment of 2009 (H1N1) pandemic influenza virus (pdH1N1) with other strains may produce more virulent and pathogenic forms, detection and their rapid characterization is critical. In this study, we reported a “one-size-fits-all” approach using a next-generation sequencing (NGS) detection platform to extensively identify influenza viral genomes for diagnosis and determination of novel virulence and drug resistance markers. A de novo module and other bioinformatics tools were used to generate contiguous sequence and identify influenza types/subtypes. Of 162 archived influenza-positive patient specimens, 161(99.4%) were positive for either influenza A or B viruses determined using the NGS assay. Among these, 135(83.3%) were A(H3N2), 14(8.6%) were A(pdH1N1), 2(1.2%) were A(H3N2) and A(pdH1N1) virus co-infections and 10(6.2%) were influenza B viruses. Of the influenza A viruses, 66.7% of A(H3N2) viruses tested had a E627K mutation in the PB2 protein, and 87.8% of the influenza A viruses contained the S31N mutation in the M2 protein. Further studies demonstrated that the NGS assay could achieve a high level of sensitivity and reveal adequate genetic information for final laboratory confirmation. The current diagnostic platform allows for simultaneous identification of a broad range of influenza viruses, monitoring emerging influenza strains with pandemic potential that facilitating diagnostics and antiviral treatment in the clinical setting and protection of the public health.

## Introduction

Influenza viruses belonging to the family Orthomyxoviridae, are classified into influenzavirus A, B, and C. While influenza A and C viruses infect humans and many other species, influenza B viruses only infect humans and seals [1,2,3]. The Influenza virus genome consists of eight negative single-stranded RNA segments encoding eleven proteins: hemagglutinin (HA), neuraminidase (NA), nucleoprotein (NP), polymerase proteins (PB1, PB2, and PA), matrix proteins (M1 and M2), and nonstructural proteins (NS1 and NS2) [4]. Based on reactivity with surface glycoproteins HA and NA, influenza A viruses are classified into eighteen HA subtypes (H1-H18) and eleven NA subtypes (N1-N11) with a total of 144 subtypes possible, while influenza B virus has no subtypes [4,5,6,7,8]. New influenza viral variants emerge either due to mutations in the virus surface glycoproteins (antigenic drift) or reassortment between viral gene segments from different strains (antigenic shift). While, seasonal influenza outbreaks are thought to arise primarily due to antigenic drift, pandemics are thought to result from complex reassortment events among swine, human, and avian reservoirs. Some of them have been reported to directly infect humans, or transmit avian influenza A viruses from domestic poultry to human [4,9,10]. In addition, influenza viruses normally circulate in pigs are called “variant” viruses when found in people. There was a large outbreak of influenza A(H3N2) variant virus (H3N2v) during the 2010–13 seasons in U.S. and a highly pathogenic avian influenza (HPAI) H5N1 virus was identified in early 2014 in North America [10]. According to a USDA report in January 21 2015, HPAI H5N1, H5N2, and H5N8 viruses, which can be potentially transmitted to domestic poultry population leading to an outbreak [11], have been detected in U.S. wild birds in the winter of 2015 [12]. Identification of H7N9, H10N8 and other viruses in humans outside the U.S. has also been reported [9,13]. Reassortment of a 2009 H1N1 pandemic influenza A virus (pdH1N1) and influenza A(H3N2) variant virus (H3N2v) with other circulating seasonal strains in the U.S. can produce virus variants with transmissibility and altered virulence for humans [14,15]. Multiple influenza types/subtypes usually circulate during each season, for example, pdH1N1 were the predominant viruses circulating in December 2013 with fewer influenza B viruses, but influenza B virus became the predominant circulating virus by late March 2014, while seasonal A(H3N2), novel H3N2v, and mixed A/B viruses infections were also identified during the season in the U.S. [16].

It was reported that rapid influenza diagnostic tests showed low and variable sensitivity [17,18] depending on the influenza A subtype [19], while some FDA-cleared diagnostic tests did not detect the novel H3N2v viruses [20]. Diagnosis of influenza infection from an unknown risk respiratory specimen involves multiple assays [17,21]. Identification of the first case of A(H5N1) virus infection in North America involved multiple laboratories [10]. Current methods that evaluate antiviral resistance require two independent tests for the S31N substitutions conferring amantadine-resistance [22,23] and the H274Y substitution conferring oseltamivir-resistance [22,24]. Conventional methods used by diagnostic or reference laboratories for characterization of novel influenza viruses are ineffective since the tests are based on a small conserved region of each HA, NA, and M genes for detection and subtyping, requires the use of many sets of primers, steps and multiple reactions. In addition, this approach may be ineffective for newly emerging influenza subtypes due to mismatch of PCR primers and culture-based genome sequencing which requires high biosafety levels, preventing its application in clinics. Next-generation sequencing (NGS) analysis has been previously reported to greatly improve manipulation procedure for identification of influenza virus strains, these studies focused on examining influenza A [25,26,27] or B [28] viruses using cultured virus [27], random [27], universal [25], and multiple [28] primers. None of these studies reported the use of a paired set of degenerate universal primers to simultaneously identify whole-genome sequences of influenza A and B viruses from a large set of clinical specimens using an NGS assay. We previously reported development of a genomic nanomicroarray and NGS combination approach for influenza virus screening and laboratory confirmation [29]. Here, we extended the NGS approach to potential application for influenza virus surveillance, whole-genome characterization of putative antiviral resistance markers and virulence factors that may confer high virulence in human hosts. We developed a novel universal RT-PCR-NGS platform by testing 162 clinical influenza-positive specimens and demonstrated a “one-size-fits-all” approach to simultaneously identify unknown influenza infections and co-infections in a single tube reaction per sample. The analytical sensitivity of the NGS platform was also evaluated in our studies.

## Materials and Methods

### Viruses, Clinical Specimens and Tests

**Fig 1:**
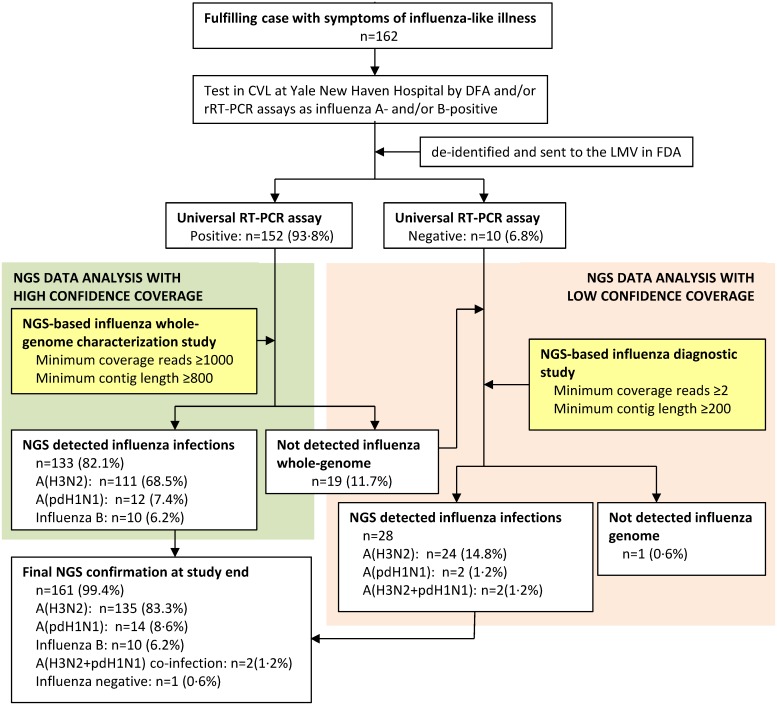
Study profile. Enrollment of 162 patients aged 20 months to 89 years with symptoms of influenza-like illness during February 19, 2011 to March 8, 2013 from over twenty cities and towns in Connecticut, and submitted to the Clinical Virology Laboratory (CVL) at Yale New Haven Hospital, New Haven, Connecticut prior to detect by universal RT-PCR and NGS assays at the Laboratory of Molecular Virology (LMV) in FDA. A total of 381 genome sequences identified from 55 specimens including A(H3N2), A(pdH1N1), and Influenza B viruses were submitted into GenBank. DFA, direct fluorescent antigen test; RT-qPCR, quantitative RT-PCR; NGS, next-generation sequencing.

Pre-titrated stocks of reference strains of influenza viruses were procured from Dr. Stephen Lindstrom (Centers for Disease Control and Prevention, CDC, Atlanta, GA) or cultured in embryonated chicken eggs at US Food and Drug Administration (FDA). Clinical nasopharyngeal swab specimens were collected from patients with symptoms of influenza-like illness, submitted during the 2010–11 and 2012–13 influenza seasons to the Clinical Virology Laboratory (CVL), Yale New Haven Hospital, New Haven, CT. These specimens were tested as requested by the patients’ physicians using a direct fluorescent antibody (DFA) immunoassay (SimulFluor Respiratory Virus Screen Reagent, Millipore, Billerica, MA) and/or by quantitative RT-PCR (RT-qPCR), as previously described [21]. A total of 162 nasopharyngeal swab specimens that tested as influenza A- and/or B-positive were de-identified and sent to the Laboratory of Molecular Virology (LMV) at FDA (Institutional Review Board (IRB) approval# Research Involving Human Subjects Committee (RIHSC):13-048B) for sequencing (Fig 1). All participants, or their parents or guardians, provided written informed consent when their specimens collected in Yale New Haven Hospital.

### RNA Extraction and Universal RT-PCR

The RNA extraction, some primers, and Reverse Transcription-PCR (RT-PCR) procedures were described previously [29] and in S1 File. According to previous reports and our testing algorithm using multiple alternative options [29,30,31,32], a new set of degenerate universal primers for RT (uniflu, 5’-IAGCARAAGC -3’) and fusion primers for PCR (unifluF, 5’-*ACGACGGGCGACA*IAGCARAAGC-3’; unifluR, 5’-*ACGACGGGCGACA*AGTAGWAACA-3’) were designed and tested. The 13bp flanking sequences italicized were added at the 5’ end of each PCR primer to enhance the annealing temperature and achieve high fidelity and yield in PCR amplification. These primers target highly conserved regions at the 5'- and 3'- terminus of each segment of influenza virus to amplify the whole-genome concomitantly, and have coverage of sequence variants for influenza A, B, and C viruses to achieve improvements in analytical sensitivity and inclusivity.

### MiSeq Sequencing

Sample preparation and bioinformatics data analysis was performed as described [29]. Briefly, the concentration of mega-amplicons were measured by using the Qubit dsDNA BR Assay System (Covaris, Woburn, MA, USA), and 1 ng of DNA product was processed for NGS sample preparation by using a Illumina Nextera XT DNA Sample Preparation Kit. Mega-amplicons for each specimen were internally marked with dual-index primers (S2 Fig). Up to 96 specimens were barcoded and run in one lane of the sequencer. NGS was performed using a MiSeq v2 kit (500 cycles) to produce 2x250 paired-end reads (Illumina, San Diego, CA). After automated cluster generation in MiSeq, the sequencing was processed and genomic sequence reads obtained.

### *De novo* Assembly Strategy

Sequencing reads of approximately 250bp in length were trimmed and the sequence data verified using FastQC software prior to *de novo* assembly. Contigs for individual gene segments from influenza viruses were generated using the *de novo* assembly module on the CLC bio software (v6.0.6, CLC bio, Cambridge, MA). The parameter for mapping reads back to contiguous was set on as similarity fraction 0.9; length fraction 0.5; mismatch cost 2; insertion cost 3 and deletion cost 3. For characterization of influenza whole-genomes with high confidence coverage, minimum contig length was set at 800bp to assemble the consensus sequences and minimum coverage was 1000 reads [33]. For diagnosis of influenza virus infections, minimum contig length used was 200bp and higher lengths for 2x reads per base in *de novo* assembly (low confidence coverage, Fig 1 and S3 Fig) [34].

### Bioinformatics Data Analysis

A master file containing all unique contigous sequences of each mega-amplicon was generated and used to perform an all-by-all blast search in Influenza Research Database (IRD, www.fludb.org), the Global Initiative on Sharing All Influenza Data (GISAID, platform.gisaid.org) and NCBI database. The match with the highest score was retained to identify the specific influenza genome. Assembled sequences were aligned by the CLUSTAL W program, and phylogenetic analysis was performed using MEGA 5 and the neighbor-joining method [35]. The amino acid sequence for each segment identified was generated and aligned using the Vector NTI Advance package (v·11·5·2, Life Technologies, Grand Island, NY). The serotype and genotype according to the genetic lineage of influenza A viruses were determined using FluGenome [36].

### Coverage of Depth and Breadth

The depth of sequence coverage (DOC) was calculated using formulae LN/G, where L is the read length, N is the number of reads and G is the theoretical genome length. The breadth of sequence coverage (BOC) was calculated as percentage of actual testing contig length (≥1000 reads or ≥30x of DOC) divided by the theoretical genome length [34,37]. Total average length of influenza A and B virus genomes was 13,588bp and 13,559bp, respectively. Representative lengths of individual gene segments from influenza A virus (A/Puerto Rico/8/1934(H1N1)) were as follows; 2341bp (PB2), 2341bp (PB1), 2233bp (PA), 1778bp (HA), 1565bp (NP), 1413bp (NA), 1027bp (M) and 890bp (NS).

### Sequence Accession Numbers

The newly reported influenza genome sequences are available in GenBank under the following accession numbers: KM654599-KM654931, and KM654933-KM654980.

## Results

### NGS Detects Influenza Whole-Genome Segments

**Fig 2:**
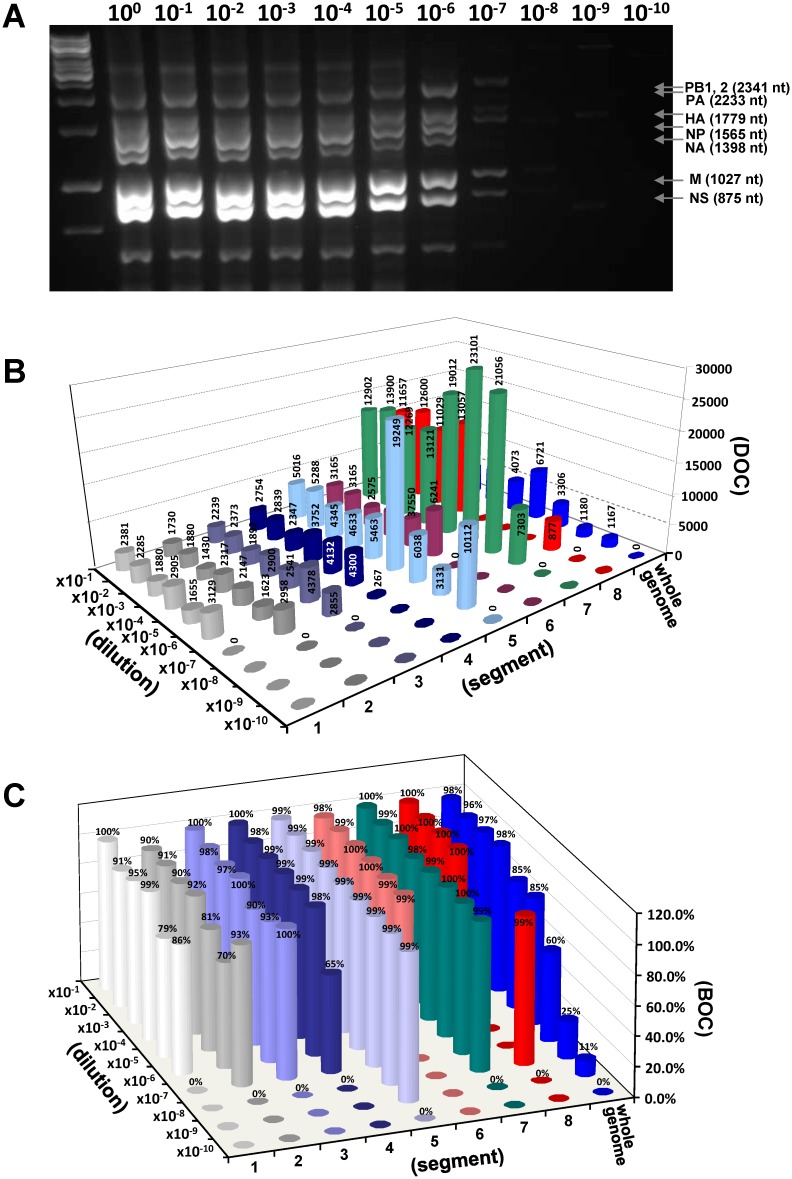
**(A) Agarose gel illustrating the sensitivity of the universal RT-PCR assay.** The picture shows PCR mega-amplicons in 2% agarose gel representing the whole-genome segments of A/Viet Nam/1203/2004(H5N1) virus in dilution series, and detection of viral RNA in a dilution of 10^−7^ (Table 1). **(B) Illustrating average depth of coverage (DOC)**. **(C) average breadth of coverage (BOC) in NGS characterization of the influenza genome**.

To determine the sensitivity of NGS detection, ten-fold serial dilutions of H5N1 viral RNA (1.8x10^9^ TCID_50_/mL) were first produced followed by RT-PCR exponential amplification for whole-genome segments. As shown in Fig 2A, 10^−7^ dilution of A(H5N1) viral RNA (1.8x10^3^ TCID_50_/mL) was detected in the RT-PCR assay shown as multiple faint visible bands. A total of ten PCR mega-amplicons from the dilutions were next tested using MiSeq, multiple contigs were finally automatically generated for each mega-amplicons, and all contig sequences were correctly identified as genome of A/Viet Nam/1203/2004(H5N1) virus. The overall average of DOC was 4,715. The average of BOC for each identified contig was 96% (≥30x depth) indicating that the near full-length genome sequence was covered (Table 1). Analysis of reads at each dilution level showed that the whole-genome DOC decreases from 4,073 in 10^−1^ dilution to 1,167 in 10^−9^ dilution (Fig 2B), and the whole-genome BOC decreases from 98% to 11% at the 10^−9^ dilution. We observed that the NP gene (segment 5) represented 99% of BOC at all detected titration points with the lowest detectable level at 10^−9^ dilution (1.8x10^1^ TCID_50_/mL) with high confidence coverage (Fig 2C).

**Table 1:** Length, reads and coverage from serial dilutions of influenza A(H5N1) virus.

gene segment (bp)		serial dilutions									
		10^−1^	10^−2^	10^−3^	10^−4^	10^−5^	10^−6^	10^−7^	10^−8^	10^−9^	10^−10^
PB2 (2341)	length (bp)	2341	2132	2230	2323	1854	2024	0	0	0	0
	reads (x10^3^)	22·3	21·4	17·6	27·2	15·5	29·3	0	0	0	0
	depth	2381	2285	1880	2905	1655	3129	0	0	0	0
	breadth	100%	91%	95%	99%	79%	86%	0%	0%	0%	0%
PB1 (2341)	length (bp)	2109	2123	2102	2158	1895	1648	2174	0	0	0
	reads (x10^3^)	16·2	17·6	13·4	21·7	20·1	15·2	27·7	0	0	0
	depth	1730	1880	1430	2317	2147	1623	2958	0	0	0
	breadth	90%	91%	90%	92%	81%	70%	93%	0%	0%	0%
PA (2233)	length (bp)	2233	2192	2171	2233	2016	2085	2224	0	0	0
	reads (x10^3^)	20	21·2	16·9	25·9	22·7	39·1	25·5	0	0	0
	depth	2239	2373	1892	2900	2541	4378	2855	0	0	0
	breadth	100%	98%	97%	100%	90%	93%	100%	0%	0%	0%
HA (1779)	length (bp)	1772	1747	1753	1757	1760	1751	1160	0	0	0
	reads (x10^3^)	19·6	20·2	16·7	26·7	29·4	30·6	1·9	0	0	0
	depth	2754	2839	2347	3752	4132	4300	267	0	0	0
	breadth	100%	98%	99%	99%	99%	98%	65%	0%	0%	0%
NP (1565)	length (bp)	1556	1556	1556	1556	1556	1554	1556	1556	1543	0
	reads (x10^3^)	31·4	33·1	27·2	29	34·2	120·5	37·8	19·6	63·3	0
	depth	5016	5288	4345	4633	5463	19249	6038	3131	10112	0
	breadth	99%	99%	99%	99%	99%	99%	99%	99%	99%	0%
NA (1398)	length (bp)	1371	1389	1398	1398	1389	1389	0	0	0	0
	reads (x10^3^)	17·7	17·7	14·4	19·3	21	34·9	0	0	0	0
	depth	3165	3165	2575	3451	3755	6241	0	0	0	0
	breadth	98%	99%	100%	100%	99%	99%	0%	0%	0%	0%
M (1027)	length (bp)	1027	1016	1027	1003	1016	1027	1027	1018	0	0
	reads (x10^3^)	53	57·1	50·4	53·9	78·1	94·9	86·5	30	0	0
	depth	12902	13900	12269	13121	19012	23101	21056	7303	0	0
	breadth	100%	99%	100%	98%	99%	100%	100%	99%	0%	0%
NS (875)	length (bp)	884	883	889	891	0	0	0	877	0	0
	reads (x10^3^)	40·8	44·1	38·6	45·7	0	0	0	14·4	0	0
	depth	11657	12600	11029	13057	0	0	0	4103	0	0
	breadth	100%	100%	100%	100%	0%	0%	0%	100%	0%	0%
Whole Genome (13559)	length (bp)	13293	13038	13126	13319	11486	11478	8141	3451	1543	0
	reads (x10^3^)	221	232·5	195·1	249·3	220·9	364·5	179·3	64	63·3	0
	depth*	4073	4287	3597	4597	4073	6721	3306	1180	1167	0
	breadth*	98%	96%	97%	98%	85%	85%	60%	25%	11%	0%
	No. of genes.	8	8	8	8	7	7	5	3	1	0

Ten-fold serial dilutions were used in titrations of influenza A(H5N1) virus to establish the analytical sensitivity by universal RT-PCR and NGS assays. Minimum contiguous length sets at 800bp to assemble the consensus sequences for characterization of each segment full-length sequence and minimum coverage is 1000 reads. A calculated whole-genome base of A/Viet Nam/1203/2004 virus is 13559bp.

* calculation for virus whole-genome.

### Molecular Confirmation of Simultaneous Detection of Influenza A and B Viruses in a Single Specimen/Test

Since some influenza related hospitalized cases were found to be associated with mixed A and B virus co-infections [1,16], analytical inclusivity studies were performed next by using a new set of degenerate universal primers to achieve improved coverage for varieties of both influenza A and B viruses. We tested A/Fujian Gulou/1896/09(H1N1), A/Perth/16/2009(H3N2), and B/Wisconsin/01/2010 reference viruses. Total RNA was extracted from spiked samples containing either individual or a mixture of two strains 1:1, and RT-PCR was performed. After the NGS test and following *de novo* assembly, eight contigs were generated for A(H1N1), A (H3N2), and B viruses, respectively. Fifteen and sixteen contigs were generated from the mixed A(H1N1), and A(H3N2) with B viruses supported by high confidence coverage, respectively (S1 Table). A validation assessment for these contigs from mixed viruses confirmed that the genome sequences are identical to the gene segments of the A(H1N1), A(H3N2), or B viruses, respectively. This is the first report of analytical validation of degenerate universal primers that can simultaneously amplify influenza A and B viruses for NGS identification and discrimination of influenza viruses in a single sample/test.

### Detection of Influenza Virus in Nasopharyngeal Swab Specimens

By using the aforementioned strategy, we next tested 162 clinical nasopharyngeal swab specimens (Fig 1) which previous detection in CVL showed 74%(120/162) DFA test positive, 60%(98/162) RT-qPCR test positive, and 6.2%(10/162) for influenza B viruses. The range of Ct values for the 162 specimens is between 17.4 and 39.2. The PCR mega-amplicons prepared for each of the clinical specimens were analyzed using MiSeq. 99.4%(161/162) specimens detected influenza viral genome (see description below, S4 Table). To simplify the table, we reported only 57 isolates with whole-genome segments including 35 A(H3N2) isolates, twelve A(pdH1N1), and ten influenza B viruses identified in NGS data analysis. The results from four different detection methods including DFA, RT-qPCR, universal RT-PCR and NGS assays were summarized in Table 2 to represent the types/subtypes for influenza viruses. Results of NGS data analysis representing full-length sequences were listed in S2 Table. The average whole-genome DOC for these viruses was between 0.1x10^3^ and 13.7x10^3^, and average whole-genome BOC was between 13% and 100% supporting high confidence coverage.

**Table 2:** Detection of influenza A and B viruses in nasopharyngeal swab specimens from naturally infected patients collected in the 2010–11 and 2012–13 seasons in Connecticut.

Patient ID	Age	Gender	Collected Date	City, State	Detection Methods			
					DFA	RT-qPCR(Ct)	Univ·PCR	NGS Verified
Flu003	9	M	12/29/2012	Hamden, CT	B	B(22·9)	++?	B
Flu053	28m.	M	12/30/2012	New Haven, CT	B	not done	+?	B
Flu096	9	F	2/5/2013	New Haven, CT	B	not done	+?	B
Flu101	72	M	3/5/2013	West Haven, CT	B	B(21·2)	++?	B
Flu103	33	F	3/7/2013	New Haven, CT	B	not done	+++?	B
Flu107	23m.	M	3/5/2013	New Haven, CT	B	not done	+?	B
Flu108	15	M	3/4/2013	Hamden, CT	B	B(18·7)	++?	B
Flu110	33	F	3/8/2013	New Haven, CT	B	B(21·4)	++?	B
Flu111	11	F	3/8/2013	East Haddam, CT	B	not done	+?	B
Flu118	3	M	1/27/2013	West Haven, CT	B	not done	++?	B
Flu042	76	M	12/28/2012	Cheshire, CT	A	not done	+++	H3N2
Flu050	79	F	12/29/2012	Essex, CT	A	A(21·1)	++++	H3N2
Flu051	36	F	12/30/2012	New Haven, CT	A	not done	++++	H3N2
Flu055	26	F	12/27/2012	East Haven, CT	A	not done	++++	H3N2
Flu056	62	F	12/30/2012	Clinton, CT	A	A(24·4)	++++	H3N2
Flu060	68	F	12/30/2012	New Haven, CT	A	not done	++++	H3N2
Flu066	29	F	12/30/2012	East Haven, CT	A	not done	++	H3N2
Flu068	84	M	12/29/2012	Hamden, CT	A	A(17·9)	++++	H3N2
Flu072	28	M	12/29/2012	New Haven, CT	A	not done	+++	H3N2
Flu074	89	M	12/29/2012	West Haven, CT	not done	A(18·0)	++++	H3N2
Flu075	47	M	12/30/2012	Killingworth, CT	A	A(19·2)	++++	H3N2
Flu085	71	M	12/31/2012	Branford, CT	A	not done	++++	H3N2
Flu086	71	M	12/31/2012	Branford, CT	A	not done	+++	H3N2
Flu090	49	M	1/1/2013	Wallingford, CT	A	A(17·4)	++++	H3N2
Flu098	82	M	2/4/2013	Milford, CT	A	A(21·8)	+++	H3N2
Flu099	44	F	1/3/2013	Madison, CT	A	A(17·9)	++++	H3N2
Flu105	20m.	F	3/4/2013	Hamden, CT	A	not done	++++	H3N2
Flu117	61	F	1/25/2013	New Haven, CT	A	not done	++++	H3N2
Flu119	6	M	1/24/2013	Shelton, CT	not done	A(20·2)	++++	H3N2
Flu122	85	F	1/27/2013	Bridgeport, CT	A	not done	++++	H3N2
Flu124	56	F	1/25/2013	New Haven, CT	A	not done	+++	H3N2
Flu135	14	F	1/28/2013	North Haven, CT	A	not done	++++	H3N2
Flu138	22m.	M	1/30/2013	New Haven, CT	A	A(19·5)	++++	H3N2
Flu139	7	M	2/1/2013	Derby, CT	A	not done	++++	H3N2
Flu140	16	M	1/29/2013	New Haven, CT	A	not done	++++	H3N2
Flu141	56	M	1/28/2013	New Haven, CT	A	not done	++++	H3N2
Flu146	44	F	2/8/2013	New Haven, CT	A	A(23·1)	++++	H3N2
Flu147	6	F	2/1/2013	New Haven, CT	A	not done	++++	H3N2
Flu159	84	M	2/10/2013	Hamden, CT	A	A(18·0)	++++	H3N2
Flu166	37	F	1/26/2013	New Haven, CT	A	A(28·8)	++++	H3N2
Flu169	24	F	1/25/2013	West Haven, CT	A	A(25·4)	++++	H3N2
Flu170	66	M	1/25/2013	West Haven, CT	not done	A(19·4)	++++	H3N2
Flu175	39	M	1/25/2013	New Haven, CT	A	A(28·5)	-	H3N2
Flu177	84	F	1/27/2013	New Haven, CT	A	A(27·5)	-	H3N2
Flu178	39	M	1/31/2013	Hamden, CT	A	not done	++++	H3N2
Flu100	39	M	2/5/2013	Meriden, CT	not done	A(23·9)	+++	pdH1N1
Flu113	42	F	1/25/2013	West Haven, CT	A	not done	++++	pdH1N1
Flu150	39	M	2/6/2013	Meriden, CT	A	not done	++++	pdH1N1
Flu161	61	F	1/31/2013	Hamden, CT	A	not done	++++	pdH1N1
Flu165	8	M	1/30/2013	North Haven, CT	A	not done	++++	pdH1N1
Flu182	55	M	4/7/2011	New Haven, CT	A	A	++++	pdH1N1
Flu183	76	F	3/7/2011	West Haven, CT	not done	A	+++	pdH1N1
Flu184	30	F	2/22/2011	Old Lyme, CT	not done	A	+++	pdH1N1
Flu186	55	M	2/19/2011	Naugatuck, CT	not done	A	+++	pdH1N1
Flu187	81	F	2/23/2011	New Haven, CT	A	A	++++	pdH1N1
Flu188	10	M	2/28/2011	Stamford, CT	A	A	++++	pdH1N1
Flu189	46	M	3/6/2011	New Haven, CT	A	A	++++	pdH1N1

Abbreviations: M, male; F, female; m., months; DFA, direct fluorescent antigen test; RT-qPCR, quantitative RT-PCR; Ct, cycle threshold; Inad, inadequate cells for DFA; A, influenza A virus; B, influenza B virus; “+”, universal PCR positive in agarose gel assay for influenza A virus; “-”, universal PCR negative in agarose gel assay; “?”, PCR positive with different pattern from influenza A virus.

### NGS Assay Shows High Detection Sensitivity

When we evaluated analytical sensitivity of the NGS method and compared it with other clinical tests (S3 Table), the criteria of minimum coverage length of 200bp and more than 2 reads was used to assemble contigs mapping to the influenza genome. Both M (278bp/810 reads) and NS (716bp/16 reads) genes were identified at the 10^−10^ dilution level in the reference H5N1 strain indicating highly sensitive detection. Likewise, we further re-analyzed ten universal RT-PCR negative specimens and 19 universal RT-PCR positive but NGS negative specimens when using the criteria of minimum coverage length of 800bp and 1000 reads (Fig 1). The NGS data showed that multiple partial influenza genomic sequences were identified in each of 28 specimens representing different influenza genomic profiles (S3 Table) and confirmed the presence of 24 A(H3N2) and two A(pdH1N1) infections. There were two cases identified as A(H3N2) and A(pdH1N1) viruses co-infections (Flu087 and Flu185). The specimen Flu175 contained full-length segments for M (1033bp/3033 reads) and NS genes (920bp/1203 reads), while Flu177 contained full-length gene sequence for the M gene (1031bp/3645 reads).

### Overall Reporting of Influenza A and B Virus Infections

A total of 27.4 million sequence reads representing a total length of 1.73 million base-pairs were obtained from 162 clinical specimens that were processed (S4 Table). Of these, approximately 26 million reads (95%) matched the influenza virus genome representing 78% of total read length with an average of 30,000 reads per contig from a total of 867 contigs. 99.4% (161/162) of specimens were identified to contain influenza genomes and their types/subtypes were determined. Among the influenza positive specimens, 83.3% (135/162) were seasonal A(H3N2), 8.6% (14/162) were A(pdH1N1), and 6.2% (10/162) were influenza B viruses. Two cases (1.2%) were identified as A(H3N2) and A(pdH1N1) virus co-infections, and influenza genome was not found in specimen Flu199. Among 123 influenza A viruses with whole-genome sequence data supported by high confidence reads (S5 Table), we obtained 83.6% (823/8 x123) contigs in total. The highest DOC and BOCs were observed for the HA, NP, NA, M, and NS genes with each demonstrating 99–100% coverage. The minimum average DOC observed for the PB1 gene was 1,467. The frequency of being detected by the RT-PCR-NGS assay was highest for gene segment M, followed by NS, NP, PA, NA, HA, PB1, and PB2. Genotype results and representative phylogenetic trees for HA, NA, and M genes are described in S1 Fig. The details of key signature amino-acid mutations from 47 selected influenza A viruses are summarized in Table 3, The molecular biomarkers for pandemic risk, drug-resistant-, transmission-associated signature mutations, and virulence factors were assessed and further described in the discussion section. We concluded that we have simultaneously and correctly identified genetically distinct lineages of influenza A(H3N2 and pdH1N1) and B viruses in clinical specimens.

**Table 3:** Comparison of the amino-acid substitutions in the proteins of 47 influenza A(H3N2, and pdH1N1) viruses.

Strain	Subtype	PB2			PB1	PA			NP	NA		M2			NS1
		199	475	627	327	100	356	409	100	119	152	11	20	31	92
Flu042	H3N2	S	M	***K***	K	***A***	***R***	***N***	V	***V***	***Y***	I	N	***N***	D
Flu050	H3N2	S	M	***K***	K	***A***	K	S	***I***	***V***	***Y***	I	N	***N***	D
Flu051	H3N2	S	M	***K***	K	***A***	***R***	S	V	***V***	***Y***	I	N	***N***	D
Flu055	H3N2	S	M	***K***	K	***A***	***R***	***N***	V	***V***	***Y***	I	N	***N***	D
Flu056	H3N2	S	M	***K***	K	***A***	K	S	***I***	***V***	***Y***	I	N	***N***	D
Flu060	H3N2	S	***L***	***K***	K	***A***	***R***	S	***I***	***V***	***Y***	I	N	***N***	D
Flu066	H3N2	S	***L***	***K***	K	***A***	***R***	S	V	***V***	***Y***	I	N	***N***	D
Flu068	H3N2	S	M	***K***	K	***A***	***R***	S	V	***V***	***Y***	I	N	***N***	D
Flu072	H3N2	S	***L***	***K***	K	***A***	***R***	S	V	***V***	***Y***	I	N	***N***	D
Flu074	H3N2	S	M	***K***	K	***A***	***R***	S	V	***V***	***Y***	I	N	***N***	D
Flu075	H3N2	S	M	***K***	K	***A***	***R***	S	V	***V***	***Y***	I	N	***N***	D
Flu085	H3N2	S	M	***K***	K	***A***	***R***	S	V	***V***	***Y***	I	N	***N***	D
Flu086	H3N2	S	M	***K***	K	***A***	***R***	S	V	***V***	***Y***	I	N	***N***	D
Flu090	H3N2	S	***L***	***K***	K	***A***	***R***	S	V	***V***	***Y***	I	N	***N***	D
Flu098	H3N2	S	M	***K***	K	***A***	***R***	S	***I***	***V***	***Y***	I	N	***N***	D
Flu099	H3N2	S	M	***K***	K	***A***	***R***	S	V	***V***	***Y***	I	N	***N***	D
Flu105	H3N2	S	***L***	***K***	K	***A***	***R***	S	V	***V***	***Y***	I	N	***N***	D
Flu117	H3N2	S	***L***	***K***	K	***A***	***R***	S	V	***V***	***Y***	I	N	***N***	D
Flu119	H3N2	S	M	***K***	K	***A***	***R***	S	V	***V***	***Y***	I	N	***N***	D
Flu122	H3N2	S	M	***K***	K	***A***	***R***	S	V	***V***	***Y***	I	N	***N***	D
Flu124	H3N2	S	M	***K***	K	***A***	***R***	S	V	***V***	***Y***	I	N	***N***	D
Flu135	H3N2	S	M	***K***	K	***A***	***R***	S	V	***V***	***Y***	I	N	***N***	D
Flu138	H3N2	S	M	***K***	K	***A***	***R***	S	V	***V***	***Y***	I	N	***N***	D
Flu139	H3N2	S	M	***K***	K	***A***	***R***	***N***	V	***V***	***Y***	I	N	***N***	D
Flu140	H3N2	S	M	***K***	K	***A***	***R***	S	V	***V***	***Y***	I	N	***N***	D
Flu141	H3N2	S	M	***K***	K	***A***	***R***	S	V	***V***	***Y***	I	N	***N***	D
Flu146	H3N2	S	M	***K***	K	***A***	***R***	S	V	***V***	***Y***	I	N	***N***	D
Flu147	H3N2	S	M	***K***	K	***A***	***R***	S	V	***V***	***Y***	I	N	***N***	D
Flu159	H3N2	S	M	***K***	K	***A***	***R***	***N***	V	***V***	***Y***	I	N	***N***	D
Flu166	H3N2	S	***L***	***K***	K	***A***	***R***	S	V	***V***	***Y***	I	N	***N***	D
Flu169	H3N2	S	M	***K***	K	***A***	***R***	S	V	***V***	***Y***	I	N	***N***	D
Flu170	H3N2	S	M	***K***	K	***A***	K	S	***I***	***V***	***Y***	I	N	***N***	D
Flu175	H3N2	S	M	***K***	K	***A***	***R***	S	V	***V***	***Y***	I	N	***N***	D
Flu177	H3N2	S	M	***K***	K	***A***	***R***	S	V	***V***	***Y***	I	N	***N***	D
Flu178	H3N2	S	M	***K***	K	***A***	***R***	S	V	***V***	***Y***	I	N	***N***	D
Flu100	pdH1N1	***A***	***L***	E	***R***	-	***R***	***N***	***I***	***S***	R	***T***	***S***	***N***	D
Flu113	pdH1N1	***A***	***L***	E	***R***	***I***	***R***	***N***	***I***	***S***	R	***T***	***S***	***N***	D
Flu150	pdH1N1	***A***	***L***	E	***R***	V	***R***	***N***	***I***	***S***	R	***T***	***S***	***N***	D
Flu161	pdH1N1	***A***	***L***	E	***R***	***I***	***R***	***N***	***I***	***S***	R	***T***	***S***	***N***	D
Flu165	pdH1N1	***A***	***L***	E	K	-	***R***	***N***	***I***	***S***	R	T	***S***	***N***	D
Flu182	pdH1N1	***A***	***L***	E	***R***	V	***R***	***N***	***I***	***S***	R	***T***	N	***N***	D
Flu183	pdH1N1	***A***	***L***	E	***R***	V	***R***	***N***	***I***	***S***	R	***T***	***S***	***N***	D
Flu184	pdH1N1	***A***	***L***	E	***R***	V	***R***	***N***	-	***S***	R	***T***	***S***	***N***	D
Flu186	pdH1N1	***A***	***L***	E	***R***	V	***R***	***N***	***I***	***S***	R	***T***	***S***	***N***	D
Flu187	pdH1N1	***A***	***L***	E	***R***	-	***R***	***N***	***I***	***S***	R	***T***	***S***	***N***	D
Flu188	pdH1N1	***A***	***L***	E	***R***	-	***R***	***N***	***I***	***S***	R	***T***	N	***N***	D
Flu189	pdH1N1	***A***	***L***	E	***R***	V	***R***	***N***	***I***	***S***	R	***T***	***S***	***N***	D
A/California/04/09	pdH1N1	***A***	***L***	E	***R***	V	***R***	***N***	V	***S***	R	***T***	***S***	***N***	D
A/Panama/07/99	H3N2	S	M	***K***	K	***A***	***R***	***N***	V	***V***	***Y***	I	N	S	D
A/Alberta/01/2014	H5N1	***A***	***L***	E	***R***	V	K	S	***R***	***S***	R	T	***S***	S	***E***

The reference sequences are based on the inspection of amino-acids found in the majority of human pdH1N1, H3N2, and H5N1 viruses. The human-specific substitution residues were highlighted in bold and italic. A dashed line indicates that a space was inserted in the sequence to preserve the alignment.

## Discussion

We performed NGS as part of a new detection platform for diagnosis of influenza virus infections using clinical specimens. A method for identification and discrimination of functional significance of diverse influenza viruses has been established. We report (i) multiplexed detection and simultaneous discrimination of influenza virus infections in nasopharyngeal swabs and identification of genetically distinct lineages of influenza A(H3N2 and pdH1N1) and B viruses in a single sequencing run, (ii) identification of influenza virus co-infections in a single specimen/test, (iii) development of new degenerate universal primer pairs for identification of influenza viruses and demonstration of highly sensitive detection of influenza virus infections using the NGS assay. We proposed a “one-size-fits-all” approach using an NGS diagnostic platform for extensive identification of a broad range of influenza virus infections including co-infections, by revealing their comprehensive genetic context and providing matrix reporting information for final sequence-based confirmation.

A total of 162 influenza-positive de-identified nasopharyngeal swab specimens collected from patients in the Connecticut were tested in a blinded fashion using the RT-PCR-NGS platform. No reassortants were found but multiple mutations were detected in the specimens tested. Sequence analysis of 123 influenza A viruses revealed that 66.7%(82/123) of A(H3N2) viruses had a single signature mutation of E627K in the PB2 protein, and 88%(108/123) of influenza A(H3N2 and pdH1N1) viruses contained the S31N mutation in the M2 protein. A mutation of PB2 E627K has been reported to confer high virulence to the virus by enhancing replication efficiency, and increasing polymerase activity and disease severity of avian influenza viruses in mammals [38]. The S31N mutation in the transmembrane region of the M2 protein confers resistance to amantadine [9,39]. The emergence of E627K(PB2) and S31N(M2) mutations raise concerns of increased human disease severity. Influenza viruses contain multi-basic amino-acid motif at the proteolytic cleavage site of HAs, which is associated with broad tissue tropism and organ dissemination and determines viral pathogenicity [40]. Investigation of HAs in tested specimens showed a single arginine that appeared at the site (PSIQSR↓G) in A(pdH1N1) and (PEKQTR↓G) in A(H3N2) viruses, suggesting that these viruses belonged to a low pathogenic strain that poses less risk to humans.

We aligned all protein sequences of 47 influenza A viruses and compared their sequences with those in humans that have been reported as amino-acid signatures in past influenza outbreaks (Table 3). The emergence of zanamivir- and oseltamivir-resistant viruses is facilitated by mutations in the NA protein, which provides a major target for developing anti-influenza drugs [41,42]. Sequence analysis revealed that the E119V signature mutations in these specimens may be susceptible to oseltamivir in A(H3N2) but not in A(pdH1N1) viruses. Three signature residues in the PA protein, PA-100A, PA-356A, and PA-409N, were reported in most H1N1, H2N2, H3N2 or H5N1 strains and could cause pandemics [43]. A mammalian-adapting V100A substitution was identified in most A(H3N2) but not in A(pdH1N1) viruses, and S409N was identified in all A(pdH1N1) and some A(H3N2) viruses, indicating their pandemic potential. NP protein plays an important role in viral RNA replication and cross-species transmission [44]. An amino-acid signature, valine at position NP-100 reported in 2009 pandemic H1N1 strain [45], was found as V100I mutation in all A(pdH1N1) and five A(H3N2) viruses, suggesting genetic changes of influenza A viruses in the 2012–13 seasons in this region. Influenza B virus is typically considered a mild disease and has been causing 20% to 50% of total influenza incidence [46]. An E105K point mutation at the NA tetramer in influenza B virus was reported leading to reduced susceptibility to NA inhibitor drugs [47]. This mutation was not identified in the B viruses tested in current study.

It has been recognized that clinical influenza tests have variable sensitivity and multiple tests may be required for accurate diagnosis. NGS analysis of influenza viruses has been previously reported [25,26,27,28]. However, the sensitivity and simultaneous detection of various influenza viruses from a large set of clinical specimens in a single test has not been reported. Our analytical sensitivity study demonstrated that NGS can identify the full-length NP gene of influenza at a 10^−9^ dilution level (1.8x10^1^ TCID_50_/mL) in the characterization study, and detect partial sequences of M and NS genes at 10^−10^ dilution level in the analytical study (S3 Table). When we performed universal RT-PCR-NGS assays in the analytical study using specimens with low virus concentrations [21], we further confirmed that multiple influenza genomic sequences were identified in each of the 28 human specimens demonstrating that NGS based diagnostics can achieve highly sensitive detection equivalent to the clinical RT-qPCR test at low virus concentrations. To determine whether the current NGS platform could detect influenza virus in different vertebrate species in addition to humans, we tested chicken specimens obtained from Egypt and identified A(H5N1) [48] and A(H9N2) (unpublished data) subtypes. Influenza C virus infection is not routinely tested in clinical settings, therefore, specimens and reference materials were not evaluated in current study.

Since the degenerate universal primers reported here target conserved sequences for influenza A, B and C viruses, it is anticipated that the current NGS based detection platform would enable detection and accurate characterization of influenza infection including novel, emerging strains and reassortants arising during outbreaks. This platform allows multiplex identification and simultaneous discrimination of functional significance in a single test and provides the whole spectrum of the influenza genome. The method described here will have significant implications from the perspective of screening, monitoring, drug resistance, and vaccine development. These features of the assay will facilitate diagnostics and antiviral treatment in the clinical setting and enhance protection of public health. With the aid of bioinformatics, mathematics and epidemiologic studies, a typical genetic matrix composition from a known, and a novel emerging influenza infection can be determined. Development of an automated assembly and analysis pipeline will help uncover new levels of innovation and efficiency of transferring raw reads to specific genomic identification which will facilitate future use of an NGS diagnostics platform for public health surveillance and in clinical microbiology laboratory [49].

## Supporting Information

S1 Fig Phylogenetic analysis of the HA, NA and M gene sequences. (TIF)

S2 Fig Experimental flow (provided in response to reviewers queries). (TIF)

S3 Fig Comparison of NGS data presented in Table 1 and S3 Table (provided in response to reviewers queries). (TIFF)

S1 File Supporting Information. (PDF)

S1 Table NGS discrimination of influenza A and B viruses in a single specimen using a set of degenerate universal primers in RT-PCR amplification. (DOC)

S2 Table de novo assembly and BLASTn analysis of 57 nasopharyngeal swab specimens. (DOC)

S3 Table Determination of analytical sensitivity using NGS sequencing-based diagnostics. (DOC)

S4 Table Summary of NGS data analysis of 162 nasopharyngeal swab specimens. (DOC)

S5 Table Summary of data analysis for characterization study of 123 influenza A viruses. (DOC)
