# Supplementary figures and images for: Sensitive Detection and Simultaneous Discrimination of Influenza A and B Viruses in Nasopharyngeal Swabs in a Single Assay Using Next-Generation Sequencing-Based Diagnostics
Source: PLoS One. 2016 Sep 22;11(9):e0163175. doi: 10.1371/journal.pone.0163175 (PMC5033603; doi:10.1371/journal.pone.0163175)

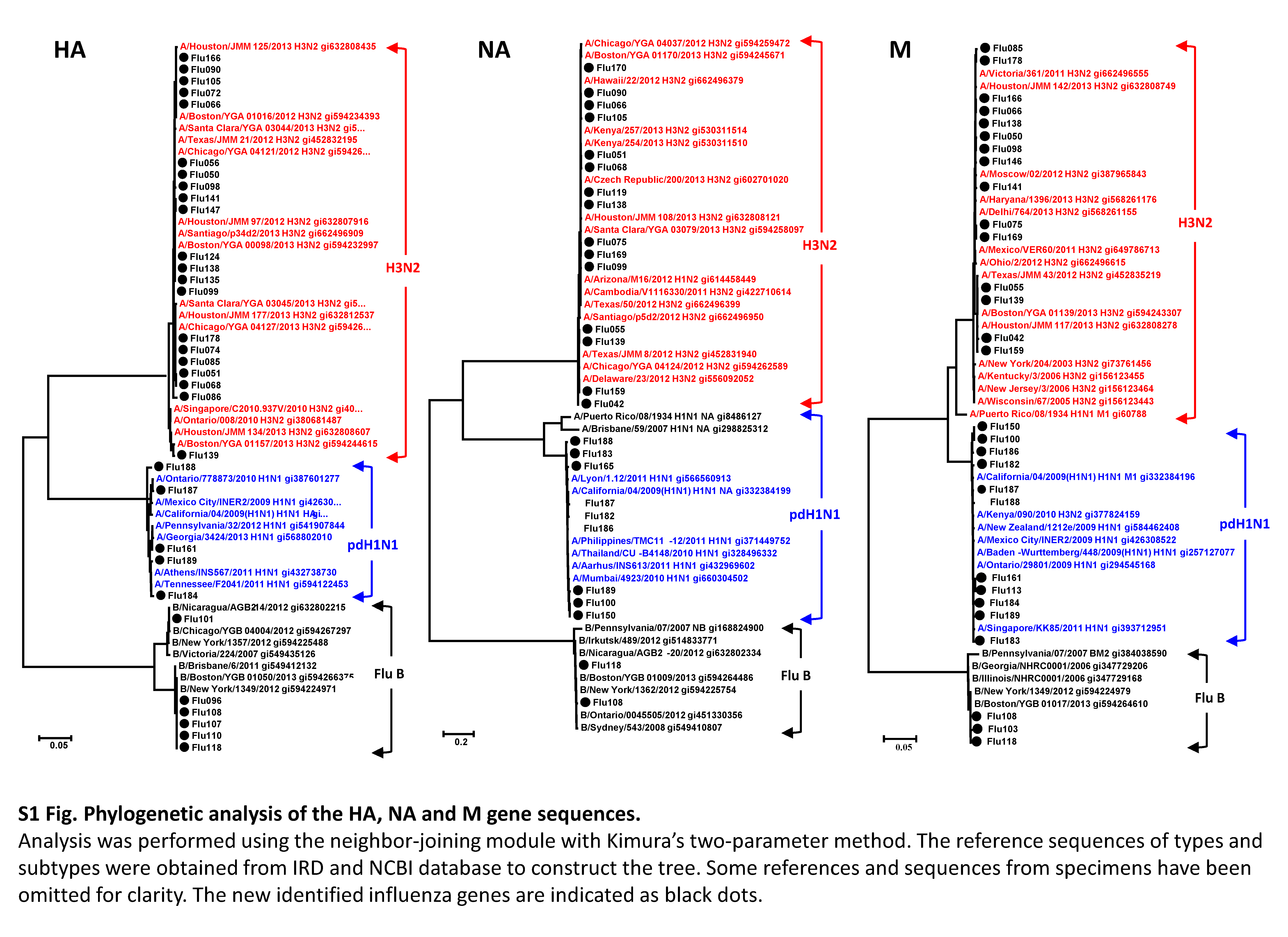

Supplement: S1 Fig — (TIF) [file pone.0163175.s001.tif]
