# Supplementary material for: Sensitive Detection and Simultaneous Discrimination of Influenza A and B Viruses in Nasopharyngeal Swabs in a Single Assay Using Next-Generation Sequencing-Based Diagnostics
Source: PLoS One. 2016 Sep 22;11(9):e0163175. doi: 10.1371/journal.pone.0163175 (PMC5033603; doi:10.1371/journal.pone.0163175)
Supplement: S1 File — (PDF) [file pone.0163175.s004.pdf]

Sensitive detection and simultaneous discrimination of influenza A and B viruses in nasopharyngeal swabs in a single assay using next-generation sequencing-based diagnostics

SUPPORTING INFORMATION

Viruses, RNA Extraction, and RT-PCR

Four influenza reference strains – A/Vietnam/1203/2004 (H5N1), A/Fujian Gulou/1896/09(H1N1), A/Perth/16/2009(H3N2), and B/Wisconsin/01/2010 were obtained from CDC, OR or cultured in embryonated chicken eggs at CBER, FDA to perform the universal RT-PCR testing and NGS assays in analytical validation study. Total viral RNA was extracted directly from allantoic fluid, cell culture supernatants, or nasopharyngeal swab specimens with QIAamp Viral RNA Mini Kit (QIAGEN, Valencia, CA). The purified RNA was quantified using a NanoDrop UV spectrometer (NanoDrop Technologies, Inc. DE). Viral RNA was first transcribed into cDNA using a degenerate universal primer uniflu in SuperScriptTM III First-Strand Synthesis System for RT-PCR (Invitrogen, CA) according to the manufacturer's instructions. For amplification of all eight gene segments, PCR was performed in a total volume of 30 µL containing 1-1.5 µL of cDNA, 15 µL of 2xPCR buffer (Extensor Hi-Fidelity ReddyMix PCR Master Mix, ABgen House, Surrey, UK), 2.5 pmol of forward and 2.5 pmol of reverse primers (unifluF and unifluR). Reaction conditions included one cycle at 94°C, 5 min, 35 cycles at 94°C, 30 sec, 50°C, 40 sec, 68°C, 2.4 min, and one cycle at 68°C, 7 min. The PCR products were electrophoresed with 2.0% agarose gel slabs in Tris-acetate-EDTA buffer to observe multiple amplicons (mega-amplicons). DNA was treated using ExoSAP-IT (Affymetrix, Santa Clara, CA) at 37 ^{o}C for 15 min and quantified using an Agilent 2100 Bioanalyzer (Agilent technologies, Inc., Wilmington, DE) prior to NGS sample preparation,

Sample Preparation for MiSeq Sequencing

The concentration of PCR mega-amplicons was measured using the Qubit dsDNA BR Assay system (Covaris, Woburn, MA) and 1 ng of DNA product was processed for NGS using a Nextera XT kit (Illumina, San Diego, CA) according to the manufacturer's instructions. The Nextera XT transposome fragmented PCR mega-amplicons into a size of about 500–700bp and added adapter sequences to their ends, allowing a 12-cycle PCR amplification to append additional unique dual indexes (i7 and i5) sequences at the end of each fragmented DNA for cluster formation. Mega-amplicons from each specimen were internally marked with these dual barcoded primers. Up to 96 specimens barcoded and over 9216 influenza virus genomes can be detected in a single sequencing run. The NGS was performed using MiSeq v2 kit (500 cycles) to produce 2x250 paired-ends read (Illumina, San Diego, CA). After automated cluster generation in MiSeq, the sequencing was processed and genomic sequence reads obtained.

Bioinformatics Data Analysis

A comprehensive database from a PCR mega-amplicons was generated for each specimen tested. Sequences were further filtered so the local database contained only one unique contig for every segment and multiple contigs were generated for one specimen. A mast FASTA file containing all unique contigous sequences of each mega-amplicon was generated and used to perform an all-by-all blast search in Influenza Research Database (IRD, www.fludb.org), the Global Initiative on Sharing All Influenza Data (GISAID, platform.gisaid.org) and NCBI database. The top-scoring blast match was selected to identify specific genome for one specimen. Assembled sequences were aligned by the CLUSTAL W program after sequencing extract, and phylogenetic analysis was performed with MEGA 5 using the neighbor-joining method (MEGA,

www.megasoftware.net). The amino acid sequence was generated and aligned using the Vector NTI Advance package (v·11·5·2, Life Technologies, Grand Island, NY). The serotype and genotype according to the genetic lineage of influenza A viruses were determined using FluGenome database (www.flugenome.org).

Genotypes and Phylogenetic Analysis Results

Genotypes of the influenza A(H3N2) viruses were determined as [A,D,B,3A,A,2A,B,1A], indicating that the H3N2 viruses circulating during the 2012-13 seasons were all from the same lineage. The genotype of the influenza A(pdH1N1) virus isolates were determined as [C,D,E,?,A,?,F,1A] (S2 Table). Phylogenetic analysis of gene segments showed that all genes closely clustered with A(H3N2), A(pdH1N1), or influenza B viruses with high bootstrap value. Most A(H3N2) virus isolates were similar to the A/Boston/YGA 00098/2013(H3N2) virus. While the A(pdH1N1) virus isolates were similar to a circulating A/Ontario/778873/2010(H1N1) virus. The ten influenza B virus isolates shared high sequence identity with B/Boston/YGB/01050/2013. Amino-acid sequences for the proteins encoded by eight each individual gene segments from different virus isolates were translated, and a total of 767 protein sequences were analyzed and compared with human influenza viral protein sequences retrieved from IRD, GISAID or NCBI databases.
