# Supplementary material for: Sensitive Detection and Simultaneous Discrimination of Influenza A and B Viruses in Nasopharyngeal Swabs in a Single Assay Using Next-Generation Sequencing-Based Diagnostics
Source: PLoS One. 2016 Sep 22;11(9):e0163175. doi: 10.1371/journal.pone.0163175 (PMC5033603; doi:10.1371/journal.pone.0163175)
Supplement: S1 Table — (DOC) [file pone.0163175.s005.doc]

**S1 Table. NGS discrimination of influenza A and B viruses in a single specimen using a set of degenerate universal primers in RT-PCR amplification.**

| **Strains** | **contigs length of segment (bp) / mapped reads (10^3^)** | | | | | | | |
| --- | --- | --- | --- | --- | --- | --- | --- | --- |
|  | **Seg· 1** | **Seg· 2** | **Seg· 3** | **Seg· 4** | **Seg· 5** | **Seg· 6** | **Seg· 7** | **Seg· 8** |
| A/FujianGulou/1896/09(H1N1) | 2111 / *47·4* | 1997 / *2·2* | 2065 / *82·6* | 1815 / *29·6* | 1551 / *45·9* | 1379 / *14·4* | 1167 / *71·8* | 400 / *1·3* |
| A/Perth/16/2009(H3N2) | 1266 / *4·2* | 2345 / *11·4* | 2232 / *98·7* | 1762 / *9·4* | 1575 / *77·9* | 1467 / *81·0* | 1013 / *97·1* | 882 / *9·7* |
| B/Wisconsin/01/2010 | 2379 / *31·2* | 2241 / *51·1* | 2328 / *72·6* | 1837 / *2·7* | 1854 / *9·7* | 572 / *0·1* | 1183 / *21·4* | 1294 / *11·0* |
| A/FujianGulou/1896/09(H1N1)  mixed with  B/Wisconsin/01/2010 | 3246 / *13·9* | 1769 / *0·06* | 2094 / *4·8* | 1613 / *0·9* | 1567 / *3·8* | 1198 / *0·3* | 1016 / *6·4* | 0 / *0* |
|  | 2480 / *14·4* | 2238 / *14·1* | 2307 / *19·9* | 1871 / *44·1* | 1850 / *3·0* | 712 / *1·5* | 1184 / *8·0* | 1087 / *39·3* |
| A/Perth/16/2009(H3N2)  mixed with  B/Wisconsin/01/2010 | 1517 / *0·5* | 1495 / *1·4* | 2235 / *18·7* | 1766 / *0·9* | 1345 / *9·8* | 1046 / *1·6* | 1257 / *20·2* | 894 / *2·2* |
|  | 2939 / *14·4* | 2241 / *24·7* | 2319 / *36·2* | 2059 / *66·9* | 1851 / *4·2* | 1218 / *1·8* | 1389 / *10·6* | 1087 / *71·7* |

*de novo* assembly module was used in CLC Genomics Workbench software package (v6·0·2) for result handing. Minimum contiguous length sets at 800bp to assemble the consensus sequences. The contigs sequence length (bp) for each segment (Seg·) supported by raw reads (Italics) indicated.
