# Supplementary material for: Sensitive Detection and Simultaneous Discrimination of Influenza A and B Viruses in Nasopharyngeal Swabs in a Single Assay Using Next-Generation Sequencing-Based Diagnostics
Source: PLoS One. 2016 Sep 22;11(9):e0163175. doi: 10.1371/journal.pone.0163175 (PMC5033603; doi:10.1371/journal.pone.0163175)
Supplement: S2 Table — (DOC) [file pone.0163175.s006.doc]

**S2 Table. *de novo* assembly and BLASTn analysis of 57 nasopharyngeal swab specimens**

| **Patient**  **ID** | **No. of contigs** | | | **Ave. reads**  **of Flu** | **contigs length of segment (bp) (*mapped reads** / DOC*)** | | | | | | | | **NGS verified virus** | | |
| --- | --- | --- | --- | --- | --- | --- | --- | --- | --- | --- | --- | --- | --- | --- | --- |
|  | **Total** | **Hum** | **Flu** |  | **PB2(2341)** | **PB1(2341)** | **PA(2233)** | **HA(1778)** | **NP(1565)** | **NA(1413)** | **M(1027)** | **NS(890)** | **wDOC*** | **wBOC** | **serotype**  **genotype** |
| Flu003 | 10 | 9 | 1 | 7632 | 0  (*0*/0) | 2038 (*7·6*/0·8) | 0  (*0*/0) | 0  (*0*/0) | 0  (*0*/0) | 0  (*0*/0) | 0  (*0*/0) | 0  (*0*/0) | 1·1 | 14% | B |
| Flu042 | 8 | 0 | 8 | 55667 | 2242 (*22·7*/2·4) | 2349 (*46·0*/4·9) | 2150 (*51·3*/5·7) | 1883 (*53·5*/7·5) | 1564 (*56·2*/9·0) | 1473 (*47·4*/8·4) | 1412 (*126·4*/30·8) | 886 (*41·9*/11·8) | 9·6 | 100% | H3N2  [A,D,B,3A,A,2A,B,1A] |
| Flu050 | 8 | 0 | 8 | 65444 | 2146 (*46·0*/4·9) | 2352 (*61·5*/6·6) | 2471 (*62·6*/7·0) | 1750 (*41·4*/5·8) | 1569 (*37·8*/6·0) | 1472 (*54·1*/9·6) | 1055 (*113·5*/27·6) | 1107 (*106·6*/29·9) | 11·3 | 100% | H3N2  [A,D,B,3A,A,2A,B,1A] |
| Flu051 | 8 | 0 | 8 | 22334 | 2348 (*16·7*/1·8) | 2345 (*19·5*/2·1) | 2024 (*18·2*/2·0) | 1763 (*20·2*/2·8) | 1573 (*10·5*/1·7) | 1469 (*18·4*/3·3) | 1029 (*40·1*/9·8) | 874 (*35·1*/9·9) | 4·0 | 99% | H3N2  [A,D,B,3A,A,2A,B,1A] |
| Flu053 | 4 | 3 | 1 | 482 | 0  (*0*/0) | 0  (*0*/0) | 1870 (*0·4*/0·1) | 0  (*0*/0) | 0  (*0*/0) | 0  (*0*/0) | 0  (*0*/0) | 0  (*0*/0) | 0·1 | 13% | B |
| Flu055 | 8 | 0 | 8 | 59250 | 2349 (*32·6*/3·5) | 2354 (*22·4*/2·4) | 2240 (*53·9*/6·0) | 1764 (*63·2*/8·9) | 1573 (*56·5*/9·0) | 1478 (*52·4*/9·3) | 1228 (*91·3*/22·2) | 875 (*101·8*/28·6) | 10·3 | 100% | H3N2  [A,D,B,3A,A,2A,B,1A] |
| Flu056 | 8 | 0 | 8 | 33763 | 2347 (*22·6*/2·4) | 2366 (*20·9*/2·2) | 2239 (*26·1*/2·9) | 1760 (*23·0*/3·2) | 1826 (*23·9*/3·8) | 1492 (*32·6*/5·8) | 1213 (*61·4*/14·9) | 1095 (*59·7*/16·8) | 5·6 | 100% | H3N2  [A,D,B,3A,A,2A,B,1A] |
| Flu060 | 8 | 0 | 8 | 52617 | 2351 (*38·5*/4·1) | 2306 (*35·8*/3·8) | 1957 (*33·1*/3·7) | 1748 (*39·7*/5·6) | 1572 (*39·0*/6·2) | 1865 (*53·2*/9·4) | 1029 (*92·7*/22·6) | 1100 (*88·9*/25·0) | 9·1 | 100% | H3N2  [A,D,B,3A,A,2A,B,1A] |
| Flu066 | 15 | 7 | 8 | 24720 | 2347 (*17·5*/1·9) | 2343 (*12·3*/1·3) | 2240 (*14·5*/1·6) | 1809  (*9·5*/1·3) | 1928 (*16·4*/2·6) | 1470 (*18·1*/3·2) | 1079 (*61·9*/15·1) | 876 (*47·7*/13·4) | 4·2 | 100% | H3N2  [A,D,B,3A,A,2A,B,1A] |
| Flu068 | 8 | 0 | 8 | 60236 | 2348 (*53·2*/5·7) | 2356 (*40·2*/4·3) | 2116 (*45·1*/5·0) | 1762 (*56·2*/7·9) | 1574 (*50·5*/8·1) | 1475 (*60·8*/10·8) | 1030 (*113·4*/27·6) | 906 (*62·4*/17·5) | 10·7 | 100% | H3N2  [A,D,B,3A,A,2A,B,1A] |
| Flu072 | 12 | 4 | 8 | 40390 | 1896 (*1·6*/0·2) | 2387 (*6·7*/0·7) | 2006 (*8·7*/1·0) | 1761  (*8·1*/1·1) | 1573 (*13·0*/2·1) | 1472 (*18·5*/3·3) | 1035 (*133·9*/32·6) | 1099 (*132·5*/37·2) | 7·3 | 97% | H3N2  [A,D,B,3A,A,2A,B,1A] |
| Flu074 | 8 | 0 | 8 | 57642 | 2140 (*11·0*/1·2) | 2067 (*14·2*/1·5) | 2033 (*34·3*/3·8) | 1761 (*69·3*/9·7) | 1562 (*25·2*/4·0) | 1385 (*34·4*/6·1) | 1212 (*131·6*/32·0) | 1238 (*141·1*/39·6) | 10·3 | 99% | H3N2  [A,D,B,3A,A,2A,B,1A] |
| Flu075 | 8 | 0 | 8 | 23363 | 2137 (*17·5*/1·9) | 2126 (*13·0*/1·4) | 2240 (*20·1*/2·3) | 1763 (*15·6*/2·2) | 1571 (*21·2*/3·4) | 1474 (*28·4*/5·0) | 1033 (*39·3*/9·6) | 913 (*31·9*/9·0) | 4·2 | 98% | H3N2  [A,D,B,3A,A,2A,B,1A] |
| Flu085 | 8 | 0 | 8 | 29381 | 2024 (*15·3*/1·6) | 2116 (*10·5*/1·1) | 1961 (*22·9*/2·6) | 1759 (*43·8*/6·2) | 1573 (*17·6*/2·8) | 1814 (*34·5*/6·1) | 1038 (*59·8*/14·6) | 888 (*30·7*/8·6) | 5·4 | 97% | H3N2  [A,D,B,3A,A,2A,B,1A] |
| Flu086 | 8 | 0 | 8 | 10044 | 2350 (*3·4*/0·4) | 2341 (*2·5*/0·3) | 2240 (*4·6*/0·5) | 1760  (*7·4*/1·0) | 1728 (*5·6*/0·9) | 1474 (*13·5*/2·4) | 1216 (*29·5*/7·2) | 888  (*13·8*/3·9) | 1·7 | 100% | H3N2  [A,D,B,3A,A,2A,B,1A] |
| Flu090 | 8 | 0 | 8 | 43701 | 2358 (*27·6*/3·0) | 2352 (*20·3*/2·2) | 2242 (*37·7*/4·2) | 1762 (*39·9*/5·6) | 1579 (*36·9*/5·9) | 1477 (*63·6*/11·2) | 1040 (*83·5*/20·3) | 890 (*40·2*/11·3) | 7·7 | 100% | H3N2  [A,D,B,3A,A,2A,B,1A] |
| Flu096 | 7 | 4 | 3 | 19446 | 0  (*0*/0) | 1856 (*5·7*/0·6) | 0  (*0*/0) | 976  (*33·7*/4·7) | 0  (*0*/0) | 0  (*0*/0) | 0  (*0*/0) | 1329 (*19·0*/4·5) | 4·2 | 29% | B |
| Flu098 | 9 | 1 | 8 | 18819 | 2241 (*6·3*/0·7) | 2262 (*16·2*/1·7) | 1801 (*17·5*/2·0) | 1762 (*16·8*/2·4) | 1572 (*9·3*/1·5) | 1475 (*9·2*/1·6) | 1031 (*63·8*/15·5) | 887  (*11·5*/3·2) | 3·5 | 96% | H3N2  [A,D,B,3A,A,2A,B,1A] |
| Flu099 | 8 | 0 | 8 | 56339 | 2044 (*27·7*/3·0) | 2355 (*13·5*/1·4) | 2242 (*47·8*/5·3) | 1763 (*66·2*/9·3) | 1578 (*44·9*/7·2) | 1472 (*80·6*/14·3) | 1030 (*111·9*/27·2) | 998 (*58·2*/16·3) | 10·0 | 99% | H3N2  [A,D,B,3A,A,2A,B,1A] |
| Flu100 | 9 | 2 | 7 | 30609 | 2139 (*1·6*/0·2) | 2160 (*1·2*/0·1) | 2103 (*5·8*/0·7) | 0  (*0*/0) | 1567 (*21·8*/3·5) | 1321 (*42·7*/7·6) | 1035 (*83·6*/20·3) | 920 (*57·6*/16·2) | 5·7 | 83% | pdH1N1  [C,D,E,?,A,?,F,1A] |
| Flu101 | 4 | 0 | 4 | 39119 | 0  (*0*/0) | 1754 (*60·6*/6·4) | 0  (*0*/0) | 999  (*41·7*/5·8) | 1478 (*1·2*/0·2) | 0  (*0*/0) | 0  (*0*/0) | 1096 (*53·0*/12·6) | 8·8 | 38% | B |
| Flu103 | 4 | 0 | 4 | 31910 | 0  (*0*/0) | 1500 (*33·8*/3·6) | 0  (*0*/0) | 0  (*0*/0) | 1673 (*15·7*/2·2) | 0  (*0*/0) | 1157  (*7·3*/1·6) | 1106 (*70·8*/16·9) | 7·0 | 38% | B |
| Flu105 | 8 | 0 | 8 | 40879 | 2320 (*19·4*/2·1) | 2349 (*24·0*/2·6) | 1902 (*35·3*/3·9) | 1762 (*31·4*/4·4) | 1574 (*30·5*/4·9) | 1473 (*59·7*/10·6) | 1034 (*89·7*/21·8) | 887 (*37·1*/10·4) | 7·4 | 98% | H3N2  [A,D,B,3A,A,2A,B,1A] |
| Flu107 | 7 | 3 | 4 | 26551 | 0  (*0*/0) | 2450 (*4·6*/0·5) | 0  (*0*/0) | 1805 (*79·0*/11·0) | 1313 (*0·9*/0·1) | 0  (*0*/0) | 0  (*0*/0) | 1101 (*21·6*/5·1) | 4·8 | 47% | B |
| Flu108 | 8 | 2 | 6 | 58642 | 0  (*0*/0) | 2047 (*16·4*/1·7) | 0  (*0*/0) | 1908 (*245·4*/34·2) | 1343 (*6·3*/0·9) | 1486 (*1·9*/0·3) | 1181  ((*2·1*/0·5) | 1117 (*79·7*/19·0) | 11·6 | 64% | B |
| Flu110 | 5 | 1 | 4 | 23174 | 0  (*0*/0) | 1762 (*7·6*/0·8) | 0  (*0*/0) | 1103 (*57·1*/7·9) | 1344 (*5·7*/0·8) | 0  (*0*/0) | 0  (*0*/0) | 1303 (*22·3*/5·3) | 5·1 | 39% | B |
| Flu111 | 3 | 0 | 3 | 1470 | 1457 (*0·6*/0·1) | 1596 (*2·6*/0·3) | 0  (*0*/0) | 0  (*0*/0) | 1021 (*1·2*/0·2) | 0  (*0*/0) | 0  (*0*/0) | 0  (*0*/0) | 0·3 | 29% | B |
| Flu113 | 7 | 0 | 7 | 11594 | 2116 (*4·8*/0·5) | 1939 (*5·5*/0·6) | 2240 (*9·5*/1·1) | 0  (*0*/0) | 1569 (*10·4*/1·7) | 1339 (*13·4*/2·4) | 1033 (*20·4*/5·0) | 891  (*17·2*/4·8) | 2·2 | 82% | pdH1N1  [C,D,E,?,A,?,F,1A] |
| Flu117 | 8 | 0 | 8 | 19502 | 2103 (*14·3*/1·5) | 2166 (*17·4*/1·9) | 1624 (*16·2*/1·8) | 1761 (*15·6*/2·2) | 1573 (*13·6*/2·2) | 1644 (*25·7*/4·6) | 1033 (*35·3*/8·6) | 1032 (*17·9*/5·0) | 3·6 | 95% | H3N2  [A,D,B,3A,A,2A,B,1A] |
| Flu118 | 6 | 0 | 6 | 26207 | 0  (*0*/0) | 1858 (*19·5*/2·1) | 0  (*0*/0) | 1880 (*80·0*/11·1) | 1436 (*13·7*/1·9) | 1140 (*3·0*/0·5) | 1186  (*5·9*/1·3) | 1106 (*35·2*/8·4) | 5·5 | 61% | B |
| Flu119 | 8 | 0 | 8 | 20694 | 1960 (*14·6*/1·6) | 2020 (*14·1*/1·5) | 1869 (*19·9*/2·2) | 1759 (*18·0*/2·5) | 1569 (*13·3*/2·1) | 1473 (*25·6*/4·5) | 1033 (*36·9*/9·0) | 889  (*23·2*/6·5) | 4·0 | 93% | H3N2  [A,D,B,3A,A,2A,B,1A] |
| Flu122 | 8 | 0 | 8 | 29534 | 2109 (*26·1*/2·8) | 2126 (*27·7*/3·0) | 2235 (*35·4*/4·0) | 1886 (*23·8*/3·4) | 1571 (*16·6*/2·7) | 1478 (*33·5*/5·9) | 1256 (*44·7*/10·9) | 888  (*28·5*/8·0) | 5·2 | 100% | H3N2  [A,D,B,3A,A,2A,B,1A] |
| Flu124 | 8 | 0 | 8 | 33191 | 2185 (*17·7*/1·9) | 2116 (*13·2*/1·4) | 1729 (*22·0*/2·5) | 1758 (*20·3*/2·9) | 1571 (*18·7*/3·0) | 1562 (*43·4*/7·7) | 1032 (*89·7*/21·8) | 885 (*40·7*/11·4) | 6·2 | 94% | H3N2  [A,D,B,3A,A,2A,B,1A] |
| Flu135 | 9 | 1 | 8 | 33393 | 1968 (*11·5*/1·2) | 2196 (*10·4*/1·1) | 2006 (*13·8*/1·5) | 1766 (*23·6*/3·3) | 1576 (*33·3*/5·3) | 1481 (*32·1*/5·7) | 1041 (*83·7*/20·4) | 891 (*58·9*/16·5) | 6·2 | 95% | H3N2  [A,D,B,3A,A,2A,B,1A] |
| Flu138 | 8 | 0 | 8 | 38711 | 2120 (*21·9*/2·3) | 2030 (*14·8*/1·6) | 2080 (*22·7*/2·5) | 1993 (*40·9*/5·8) | 1649 (*37·4*/6·0) | 1530 (*42·9*/7·6) | 1187 (*65·6*/16·0) | 1058 (*63·4*/17·8) | 6·8 | 100% | H3N2  [A,D,B,3A,A,2A,B,1A] |
| Flu139 | 8 | 0 | 8 | 49500 | 2159 (*25·8*/2·8) | 2028 (*22·7*/2·4) | 1890 (*39·4*/4·4) | 1803 (*66·7*/9·4) | 1577 (*46·2*/7·4) | 1491 (*56·4*/10·0) | 1031 (*60·5*/14·7) | 885 (*78·3*/22·0) | 9·2 | 95% | H3N2  [A,D,B,3A,A,2A,B,1A] |
| Flu140 | 8 | 0 | 8 | 34766 | 2090 (*7·7*/0·8) | 2349 (*11·7*/1·2) | 2241 (*13·7*/1·5) | 1761 (*26·1*/3·7) | 1576 (*32·4*/5·2) | 1473 (*40·2*/7·1) | 1036 (*75·9*/18·5) | 892 (*70·4*/19·8) | 6·2 | 99% | H3N2  [A,D,B,3A,A,2A,B,1A] |
| Flu141 | 8 | 0 | 8 | 49822 | 2106 (*36·7*/3·9) | 2353 (*29·5*/3·1) | 1892 (*38·3*/4·3) | 1805 (*47·9*/6·7) | 1711 (*51·4*/8·2) | 1476 (*55·2*/9·8) | 1036 (*77·4*/18·8) | 890 (*62·2*/17·5) | 9·0 | 98% | H3N2  [A,D,B,3A,A,2A,B,1A] |
| Flu146 | 8 | 0 | 8 | 36829 | 2062 (*22·2*/2·4) | 1835 (*14·7*/1·6) | 1836 (*21·1*/2·4) | 2005 (*31·8*/4·5) | 1571 (*35·4*/5·6) | 1474 (*40·7*/7·2) | 1079 (*62·6*/15·2) | 899 (*66·2*/18·6) | 6·9 | 94% | H3N2  [A,D,B,3A,A,2A,B,1A] |
| Flu147 | 8 | 0 | 8 | 39699 | 2076 (*20·7*/2·2) | 2112 (*19·7*/2·1) | 2026 (*24·3*/2·7) | 1765 (*36·1*/5·1) | 1633 (*40·4*/6·5) | 1468 (*47·5*/8·4) | 1036 (*64·8*/15·8) | 891 (*64·3*/18·1) | 7·3 | 96% | H3N2  [A,D,B,3A,A,2A,B,1A] |
| Flu150 | 7 | 0 | 7 | 26500 | 2226 (*8·4*/0·9) | 2049 (*10·5*/1·1) | 2043 (*12·4*/1·4) | 0  (*0*/0) | 1570 (*35·8*/5·7) | 1441 (*27·9*/4·9) | 1036 (*46·4*/11·3) | 952 (*44·0*/12·4) | 4·9 | 83% | pdH1N1  [C,D,E,?,A,?,F,1A] |
| Flu159 | 8 | 0 | 8 | 74442 | 1999 (*40·4*/4·3) | 2354 (*37·9*/4·0) | 1884 (*73·5*/8·2) | 1860 (*83·6*/11·8) | 1575 (*90·0*/14·4) | 1478 (*86·8*/15·4) | 1035 (*125·3*/30·5) | 885 (*58·1*/16·3) | 13·7 | 96% | H3N2  [A,D,B,3A,A,2A,B,1A] |
| Flu161 | 8 | 0 | 8 | 30433 | 1798 (*5·5*/0·6) | 1838 (*12·7*/1·4) | 1509 (*13·1*/1·5) | 1628 (*34·7*/4·9) | 1572 (*40·6*/6·5) | 1296 (*31·9*/5·6) | 1034 (*54·8*/13·3) | 957 (*50·2*/14·1) | 6·3 | 86% | pdH1N1  [C,D,E,?,A,?,F,1A] |
| Flu165 | 8 | 0 | 8 | 46818 | 2054 (*12·0*/1·3) | 1956 (*19·1*/2·0) | 1462 (*5·9*/0·7) | 1705 (*41·5*/5·8) | 1702 (*75·3*/12·0) | 1507 (*41·0*/7·3) | 1032 (*100·6*/24·5) | 892 (*79·2*/22·2) | 9·1 | 91% | pdH1N1  [C,D,E,?,A,?,F,1A] |
| Flu166 | 8 | 0 | 8 | 44513 | 2352 (*23·0*/2·5) | 2352 (*12·3*/1·3) | 2292 (*24·5*/2·7) | 1764 (*36·5*/5·1) | 1731 (*44·5*/7·1) | 1476 (*48·8*/6·8) | 1031 (*87·4*/21·3) | 1116 (*79·2*/22·3) | 7·6 | 100% | H3N2  [A,D,B,3A,A,2A,B,1A] |
| Flu169 | 8 | 0 | 8 | 38691 | 2193 (*20·6*/2·2) | 2350 (*6·1*/0·7) | 2242 (*21·9*/2·5) | 1765 (*42·0*/5·9) | 1574 (*35·7*/5·7) | 1487 (*46·1*/8·2) | 1086 (*83·2*/20·3) | 891 (*53·9*/15·1) | 6·8 | 100% | H3N2  [A,D,B,3A,A,2A,B,1A] |
| Flu170 | 8 | 0 | 8 | 30364 | 2174 (*24·3*/2·6) | 2353 (*6·1*/0·6) | 2240 (*23·6*/2·6) | 1749 (*29·7*/4·2) | 1573 (*24·9*/4·0) | 1474 (*32·8*/5·8) | 1034 (*54·5*/13·3) | 891 (*47·1*/13·2) | 5·4 | 99% | H3N2  [A,D,B,3A,A,2A,B,1A] |
| Flu175 | 8 | 6 | 2 | 2120 | 0 (*0*/0) | 0 (*0*/0) | 0 (*0*/0) | 0 (*0*/0) | 0 (*0*/0) | 0 (*0*/0) | 1033 (*3·0*/0·7) | 920 (*1·2*/0·3) | 0·7 | 14% | H3N2  [A,D,B,3A,A,2A,B,1A] |
| Flu177 | 4 | 3 | 1 | 3649 | 0  (*0*/0) | 0  (*0*/0) | 0  (*0*/0) | 0  (*0*/0) | 0  (*0*/0) | 0  (*0*/0) | 1031  (*3·6*/0·9) | 0  (*0*/0) | 1·1 | 8% | H3N2  [A,D,B,3A,A,2A,B,1A] |
| Flu178 | 8 | 0 | 8 | 37299 | 1895 (*3·3*/0·4) | 1997 (*3·0*/0·3) | 1682 (*5·9*/0·7) | 1811 (*27·7*/3·9) | 1570 (*14·6*/2·3) | 1457 (*26·9*/4·8) | 1033 (*112·0*/27·3) | 891 (*104·9*/29·5) | 7·3 | 91% | H3N2  [A,D,B,3A,A,2A,B,1A] |
| Flu182 | 9 | 1 | 8 | 29500 | 2324 (*2·6*/0·3) | 2328 (*2·6*/0·3) | 2157 (*3·2*/0·4) | 1778 (*30·1*/4·2) | 1571 (*47·8*/7·6) | 1568 (*21·6*/3·8) | 1033 (*72·2*/17·6) | 892 (*55·9*/15·7) | 5·2 | 100% | pdH1N1  [C,D,E,?,A,?,F,1A] |
| Flu183 | 7 | 0 | 7 | 32090 | 2045 (*2·9*/0·3) | 2079 (*1·8*/0·2) | 2240 (*7·6*/0·9) | 0  (*0*/0) | 1577 (*51·2*/8·2) | 1467 (*35·1*/6·2) | 1031 (*64·0*/15·6) | 886 (*62·0*/17·4) | 6·0 | 83% | pdH1N1  [C,D,E,?,A,?,F,1A] |
| Flu184 | 8 | 4 | 4 | 3290 | 0  (*0*/0) | 0  (*0*/0) | 0  (*0*/0) | 1780  (*0·8*/0·1) | 1355 (*3·9*/0·6) | 1460 (*1·0*/0·2) | 1248  (*7·3*/1·8) | 0  (*0*/0) | 0·7 | 43% | pdH1N1  [?,?,?,1A,A,?,F,?] |
| Flu186 | 13 | 10 | 3 | 18238 | 0  (*0*/0) | 0  (*0*/0) | 0  (*0*/0) | 0  (*0*/0) | 1579 (*19·0*/3·0) | 1463 (*5·2*/0·9) | 1037 (*30·6*/7·4) | 0  (*0*/0) | 4·0 | 30% | pdH1N1  [?,?,?,?,A,?,F,?] |
| Flu187 | 11 | 3 | 8 | 21780 | 2028 (*1·7*/0·2) | 2208 (*10·4*/1·1) | 1835 (*4·4*/0·5) | 1805 (*18·4*/2·6) | 1572 (*31·0*/4·9) | 1464 (*14·3*/2·5) | 1033 (*52·3*/12·7) | 945 (*41·8*/11·7) | 4·1 | 95% | pdH1N1  [C,D,E,?,A,?,F,1A] |
| Flu188 | 8 | 0 | 8 | 28382 | 2005 (*5·2*/0·6) | 2051 (*4·1*/0·4) | 1451 (*2·8*/0·3) | 1792 (*31·2*/4·4) | 1569 (*46·9*/7·5) | 1464 (*26·4*/4·7) | 1038 (*63·4*/15·4) | 965 (*47·2*/13·3) | 5·5 | 91% | pdH1N1  [C,D,E,?,A,?,F,1A] |
| Flu189 | 8 | 0 | 8 | 30545 | 2009 (*4·9*/0·5) | 1703 (*1·6*/0·2) | 1695 (*3·0*/0·3) | 1505 (*25·1*/3·5) | 1570 (*50·5*/8·1) | 1430 (*28·9*/5·1) | 1038 (*71·7*/17·5) | 889 (*58·7*/16·5) | 6·2 | 87% | pdH1N1  [C,D,E,?,A,?,F,1A] |

The contig sequences (bp) were supported by reads (Italics) and DOC (underline). DOC, average depth of coverage; wDOC, DOC for virus whole-genome; BOC, average breadth of coverage; wBOC, BOC for virus whole-genome.

* x10^3^
