# Supplementary material for: Sensitive Detection and Simultaneous Discrimination of Influenza A and B Viruses in Nasopharyngeal Swabs in a Single Assay Using Next-Generation Sequencing-Based Diagnostics
Source: PLoS One. 2016 Sep 22;11(9):e0163175. doi: 10.1371/journal.pone.0163175 (PMC5033603; doi:10.1371/journal.pone.0163175)
Supplement: S3 Table — (DOC) [file pone.0163175.s007.doc]

**S3 Table. Determination of analytical sensitivity using NGS sequencing-based diagnostics**

| strains | DFA | RT-qPCR (Ct.) | Univ.RT-PCR | *de novo* assembly of segment contigs (bp / reads) | | | | | | | | | | |
| --- | --- | --- | --- | --- | --- | --- | --- | --- | --- | --- | --- | --- | --- | --- |
|  |  |  |  | No. genes (*de*.800^a^) | No. genes (*de*.200^b^) | NGS verified | PB2(2341) | PB1(2341) | PA(2233) | HA(1778) | NP(1565) | NA(1413) | M(1027) | NS(890) |
| x 10^-7*^ | n.d. | n.d. | + | 5 | 7 | H5N1 | 0 / *0* | 2179 / *27678* | 2232 / *25468* | 1160 / *1859* | 1637 / *37912* | 1359 / *126* | 1074 / *85752* | 405 / *152* |
| x 10^-8*^ | n.d. | n.d. | - | 3 | 7 | H5N1 | 0 / *0* | 275 / *1533* | 430 / *6* | 365 / *3* | 1558 / *19501* | 330 / *4* | 1020 / *30020* | 888 / *14365* |
| x 10^-9*^ | n.d. | n.d. | - | 1 | 6 | H5N1 | 0 / *0* | 800 / *4* | 401 / *5* | 566 / *4* | 1543 / *63343* | 0 / *0* | 1029 / *573* | 490 / *18* |
| x 10^-10*^ | n.d. | n.d. | - | 0 | 2 | H5N1 | 0 / *0* | 0 / *0* | 0 / *0* | 0 / *0* | 0 / *0* | 0 / *0* | 278 / *810* | 716 / *16* |
| Flu003 | B | B(22·9) | ++? | 1 | 5 | B | 0 / *0* | 1119 / *7484* | 2264 / *198* | 336 / *815* | 0 / *0* | 0 / *0* | 869 / *55* | 1098 / *525* |
| Flu016 | n.d. | A(39·2) | + | 0 | 2 | H3N2 | 0 / *0* | 0 / *0* | 0 / *0* | 0 / *0* | 841 / *6* | 0 / *0* | 970 / 27 | 0 / *0* |
| Flu057 | A | n.d. | - | 0 | 3 | H3N2 | 0 / *0* | 660 / *9* | 454 / *12* | 1484 / *76* | 0 / *0* | 0 / *0* | 0 / *0* | 0 / *0* |
| Flu071 | A | n.d. | - | 0 | 4 | H3N2 | 1806 / *29* | 0 / *0* | 1363 / *24* | 950 / *197* | 1098 / *51* | 0 / *0* | 0 / *0* | 0 / *0* |
| Flu079 | n.d. | A(37·8) | - | 0 | 2 | H3N2 | 0 / *0* | 0 / *0* | 788 / *7* | 0 / *0* | 466 / *14* | 0 / *0* | 0 / *0* | 0 / *0* |
| Flu081 | A | A(29·8) | - | 0 | 4 | H3N2 | 0 / *0* | 0 / *0* | 2050 / *215* | 1752 / *493* | 1563 / *259* | 0 / *0* | 0 / *0* | 897 / *598* |
| Flu087 | n.d. | A(30·8) | - | 0 | 7 | pdH1N1 | 0 / *0* | 505 / *4* | 328 / *6* | 901 / *31* | 0 / *0* | 747 / *20* | 340 / *8* | 0 / *0* |
|  |  |  |  |  |  | H3N2 | 0 / *0* | 791 / *10* | 0 / *0* | 718 / *8* | 0 / *0* | 0 / *0* | 0 / *0* | 0 / *0* |
| Flu094 | A | n.d. | - | 0 | 3 | H3N2 | 279 / *18* | 0 / *0* | 276 / *8* | 0 / *0* | 533 / *7* | 0 / *0* | 0 / *0* | 0 / *0* |
| Flu112 | n.d. | A(37·5) | - | 0 | 4 | H3N2 | 0 / *0* | 606 / *9* | 836 / *7* | 587 / *9* | 0 / *0* | 390 / *4* | 0 / *0* | 0 / *0* |
| Flu126 | n.d. | A(33·5) | ++ | 0 | 3 | H3N2 | 550 / *4* | 0 / *0* | 0 / *0* | 0 / *0* | 985 / *18* | 0 / *0* | 0 / *0* | 806 / *76* |
| Flu127 | A | n.d. | ++ | 0 | 3 | H3N2 | 0 / *0* | 0 / *0* | 0 / *0* | 0 / *0* | 1568 / *501* | 0 / *0* | 1031 / *4024* | 970 / *2807* |
| Flu128 | n.d. | A(35·9) | + | 0 | 4 | H3N2 | 0 / *0* | 255 / *2* | 501 / *5* | 0 / *0* | 0 / *0* | 0 / *0* | 455 / *11* | 800 / *16* |
| Flu129 | n.d. | A(36·0) | ++ | 0 | 2 | H3N2 | 0 / *0* | 0 / *0* | 479 / *3* | 0 / *0* | 0 / *0* | 0 / *0* | 0 / *0* | 751 / *7* |
| Flu131 | n.d. | A(35·7) | ++ | 0 | 2 | H3N2 | 0 / *0* | 0 / *0* | 0 / *0* | 0 / *0* | 0 / *0* | 0 / *0* | 692 / *17* | 1229 / *13* |
| Flu133 | n.d. | A(33·1) | ++ | 0 | 2 | H3N2 | 0 / *0* | 0 / *0* | 0 / *0* | 0 / *0* | 0 / *0* | 0 / *0* | 954 / *28* | 620 / *16* |
| Flu137 | n.d. | A(32·8) | + | 0 | 2 | pdH1N1 | 0 / *0* | 0 / *0* | 0 / *0* | 275 / *4* | 0 / *0* | 0 / *0* | 0 / *0* | 236 / *2* |
| Flu142 | n.d. | A(34·3) | + | 0 | 5 | H3N2 | 0 / *0* | 302 / *7* | 646 / *19* | 744 / *14* | 428 / *15* | 0 / *0* | 892 / *243* | 0 / *0* |
| Flu151 | n.d. | A(36·0) | +++ | 0 | 2 | H3N2 | 0 / *0* | 0 / *0* | 0 / *0* | 760 / *7* | 0 / *0* | 0 / *0* | 553 / *13* | 0 / *0* |
| Flu152 | A | A(37·7) | ++ | 0 | 2 | H3N2 | 0 / *0* | 0 / *0* | 999 / *7* | 371 / *8* | 0 / *0* | 0 / *0* | 0 / *0* | 0 / *0* |
| Flu167 | n.d. | A(33·1) | + | 0 | 3 | H3N2 | 2310 / *81* | 0 / *0* | 0 / *0* | 0 / *0* | 0 / *0* | 0 / *0* | 1034 / *439* | 803 / *621* |
| Flu168 | A | n.d. | +++ | 0 | 4 | H3N2 | 2318 / *95* | 0 / *0* | 742 / *12* | 343 / *5* | 0 / *0* | 0 / *0* | 0 / *0* | 1038 / *364* |
| Flu172 | n.d. | A(30·5) | +++ | 0 | 4 | H3N2 | 683 / *5* | 0 / *0* | 649 / *5* | 676 / *8* | 0 / *0* | 0 / *0* | 0 / *0* | 860 / *189* |
| Flu175 | A | A(28·5) | - | 2 | 5 | H3N2 | 1840 / *39* | 0 / *0* | 0 / *0* | 1322 / *115* | 0 / *0* | 1575 / *162* | 1033 / *3033* | 920 / *1203* |
| Flu177 | A | A(27·5) | - | 1 | 6 | H3N2 | 203 / *1322* | 0 / *0* | 289 / *249* | 298 / *11* | 0 / *0* | 1117 / *141* | 1031 / *3645* | 565 / *1451* |
| Flu180 | n.d. | A(37·1) | + | 0 | 3 | H3N2 | 0 / *0* | 0 / *0* | 0 / *0* | 0 / *0* | 304 / *9* | 881 / *61* | 0 / *0* | 899 / *630* |
| Flu185 | n.d. | A^c^ | +++ | 0 | 8 | pdH1N1 | 465 / *28* | 0 / *0* | 0 / *0* | 1120 / *104* | 1415 / *161* | 0 / *0* | 932 / *491* | 0 / *0* |
|  |  |  |  |  |  | H3N2 | 0 / *0* | 319 / *3* | 0 / *0* | 0 / *0* | 525 / *7* | 0 / *0* | 936 / *99* | 337 / *16* |
| Flu190 | A | n.d. | ++ | 0 | 3 | pdH1N1 | 0 / *0* | 0 / *0* | 0 / *0* | 287 / *10* | 0 / *0* | 0 / *0* | 399 / *5* | 878 / *118* |
| Flu192 | n.d. | n.d. | ++ | 0 | 1 | H3N2 | 0 / *0* | 0 / *0* | 0 / *0* | 0 / *0* | 0 / *0* | 0 / *0* | 0 / *0* | 693 / *32* |
| Flu193 | n.d. | n.d. | ++ | 0 | 4 | H3N2 | 0 / *0* | 0 / *0* | 0 / *0* | 1397 / *131* | 0 / *0* | 1185 / *90* | 1069 / *446* | 867 / *255* |

Ten-fold serial dilutions were used in titrations of influenza A/Viet Nam/1203/2004(H5N1) virus. The contigs sequences (bp) determined in the characterization studies for whole genome sequence analyses were underlined. Reads indicated in italic. DFA, direct fluorescent antigen test; RT-qPCR, quantitative RT-PCR; Ct, cycle threshold; n.d., not done; A, influenza A virus; B, influenza B virus; “+ or -”, universal RT-PCR positive or negative in agarose gel assay for influenza A virus. “?”, PCR positive with different pattern from influenza A virus.

^a^ Number of segments detected, minimum contiguous length sets at 800bp to assemble the consensus sequences for whole influenza genome characterization, and minimum coverage sets at 1000 reads

^b^ Number of segments detected, minimum contiguous length sets on 200bp to assemble the consensus sequences for detection of influenza viral genome.

^c^ No record for Ct value.

^*^ A/VietNam/1203/2004(H5N1) serial dilution.
