# Supplementary material for: Sensitive Detection and Simultaneous Discrimination of Influenza A and B Viruses in Nasopharyngeal Swabs in a Single Assay Using Next-Generation Sequencing-Based Diagnostics
Source: PLoS One. 2016 Sep 22;11(9):e0163175. doi: 10.1371/journal.pone.0163175 (PMC5033603; doi:10.1371/journal.pone.0163175)
Supplement: S4 Table — (DOC) [file pone.0163175.s008.doc]

**S4 Table. Summary of NGS data analysis of 162 nasopharyngeal swab specimens**

|  | **No. of specimens** | **Mapped reads** | **Reads length (bp)** | **No. of contigs** | **Ave. reads/contig** |
| --- | --- | --- | --- | --- | --- |
| Univ. RT-PCR |  |  |  |  |  |
| positive | 152 (93.8%) | - | - | - | - |
| negative | 10 (6·2%) | - | - | - | - |
| NGS |  |  |  |  |  |
| NGS total counts | 162 | 27,351,821 | 1,725,994 | 1,284 | 21,302 |
| verified influenza infections | 161 (99·4%) | 25,962,370 (95%) | 1,354,791 (78%) | 867 | 29,945 |
| confirmed A(H3N2) | 135 (83.3%) | - | - | - | - |
| confirmed A(pdH1N1) | 14 (8·6%) | - | - | - | - |
| confirmed A(pdH1N1+H3N2) | 2 (1·2%) | - | - | - | - |
| confirmed influenza B virus | 10 (6·2%) | - | - | - | - |
| negative | 1 (0·6%) | - | - | - | - |

Univ. RT-PCR, universal RT-PCR for detection of influenza virus infections.
