# Supplementary material for: Sensitive Detection and Simultaneous Discrimination of Influenza A and B Viruses in Nasopharyngeal Swabs in a Single Assay Using Next-Generation Sequencing-Based Diagnostics
Source: PLoS One. 2016 Sep 22;11(9):e0163175. doi: 10.1371/journal.pone.0163175 (PMC5033603; doi:10.1371/journal.pone.0163175)
Supplement: S5 Table — (DOC) [file pone.0163175.s009.doc]

**S5 Table**. **Summary of data analysis for characterization study of 123 influenza A viruses**

| **Gene segment(bp)** | **PB2(2341)** | **PB1(2341)** | **PA(2233)** | **HA(1778)** | **NP(1565)** | **NA(1413)** | **M(1027)** | **NS(890)** |
| --- | --- | --- | --- | --- | --- | --- | --- | --- |
| No. of segment identified | 100 (81%) | 93 (76%) | 104 (85%) | 97 (79%) | 104 (85%) | 102 (83%) | 118 (96%) | 105 (85%) |
| Total No. of length (bp) | 203,739 | 195,163 | 202,210 | 170,740 | 166,331 | 150,658 | 127,939 | 98,102 |
| Ave. length of segment (bp) | 2,037 | 2,099 | 1,944 | 1,760 | 1,599 | 1,477 | 1,084 | 934 |
| Total No. of sequence reads | 635,632 | 599,337 | 628,978 | 507,020 | 489,499 | 439,160 | 372,930 | 284,810 |
| Ave. reads per segment | 15,750 | 13,733 | 20,554 | 28,715 | 27,496 | 31,236 | 59,335 | 45,097 |
| DOC | 1,682 | 1,467 | 2,301 | 4,038 | 4,392 | 5,527 | 14,444 | 12,668 |
| BOC | 87% | 90% | 87% | 99% | 100% | 100% | 100% | 100% |

DOC, average depth of coverage; BOC, average breadth of coverage.
